# Supplementary material for: ACLP Activates Cancer-Associated Fibroblasts and Inhibits CD8+ T-Cell Infiltration in Oral Squamous Cell Carcinoma
Source: Cancers (Basel). 2023 Aug 28;15(17):4303. doi: 10.3390/cancers15174303 (PMC10486706; doi:10.3390/cancers15174303)
Supplement: Supplementary file 1 [file cancers-15-04303-s001.zip › cancers-2563547-supplementary.pdf]

Figure S1

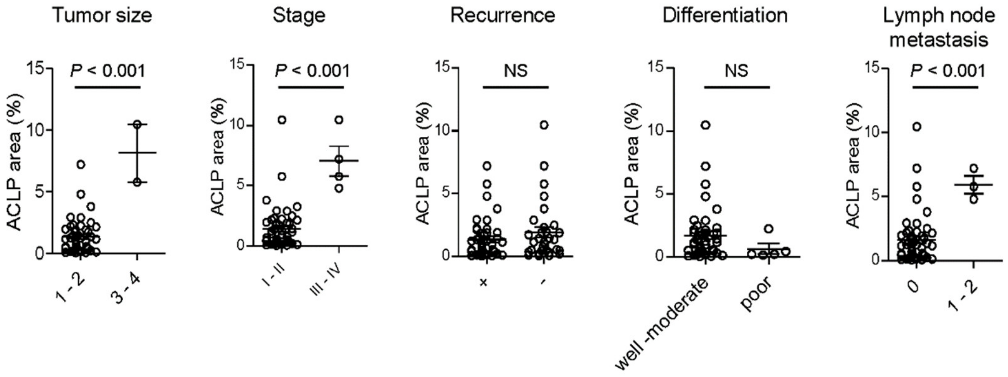

Figure S1

Correlations between the extent of ACLP-positive areas and clinicopathological characteristics in an independent set of primary OSCCs (n = 49).

Figure S2

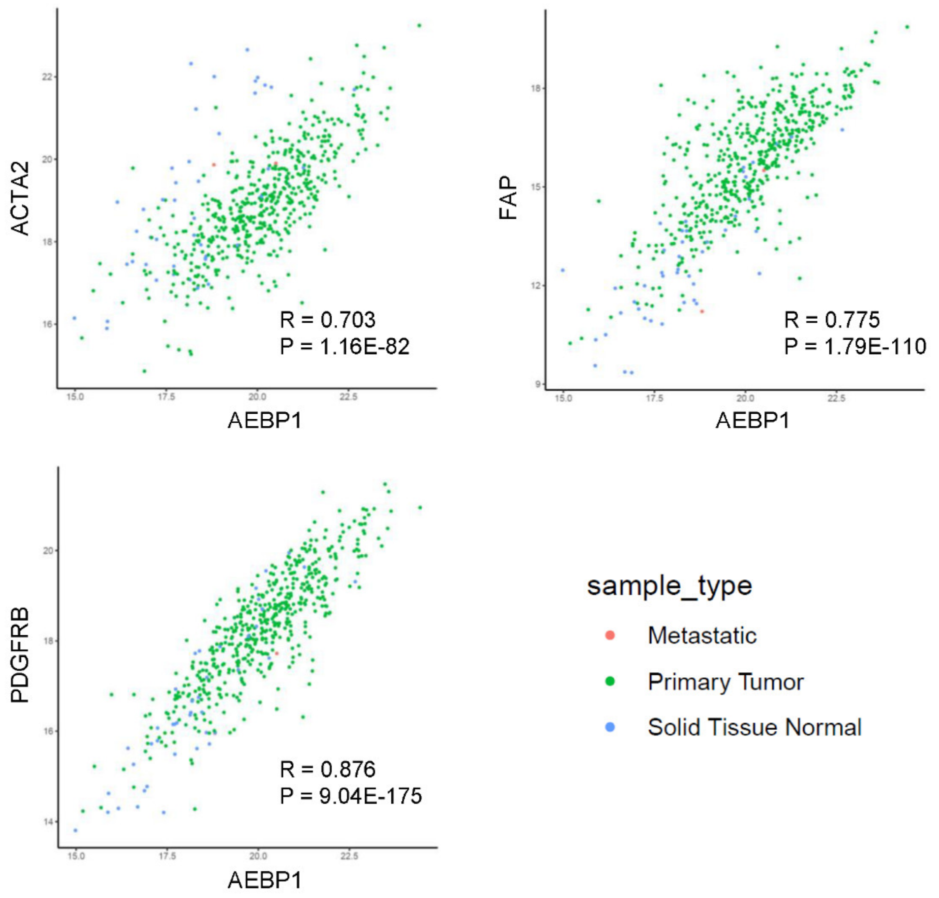

Figure S2

Correlations between the mRNA expression levels of CAF markers (*ACTA2*, *FAP* and *PDGFRB*) and those of *AEBP1* in primary HNSCCs in a dataset from The Cancer Genome Atlas (TCGA).

Figure S3

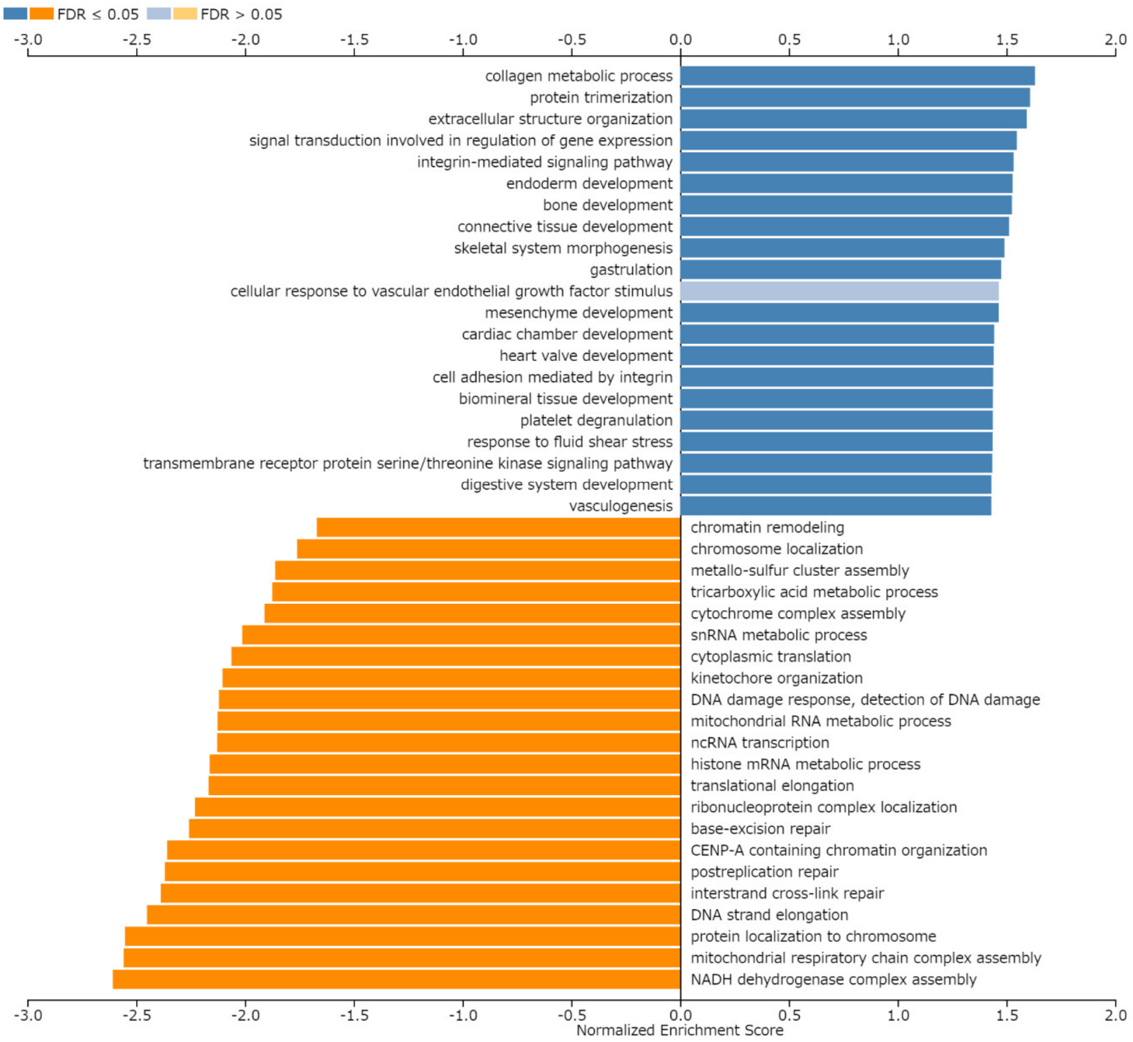

Figure S3

Gene Ontology analysis of genes correlated positively (upper) or negatively (lower) with *AEBP1* using RNA-seq data obtained from primary HNSCCs in a dataset from The Cancer Genome Atlas (TCGA). The analysis was performed using LinkedOmics (http://linkedomics.org).

Figure S4

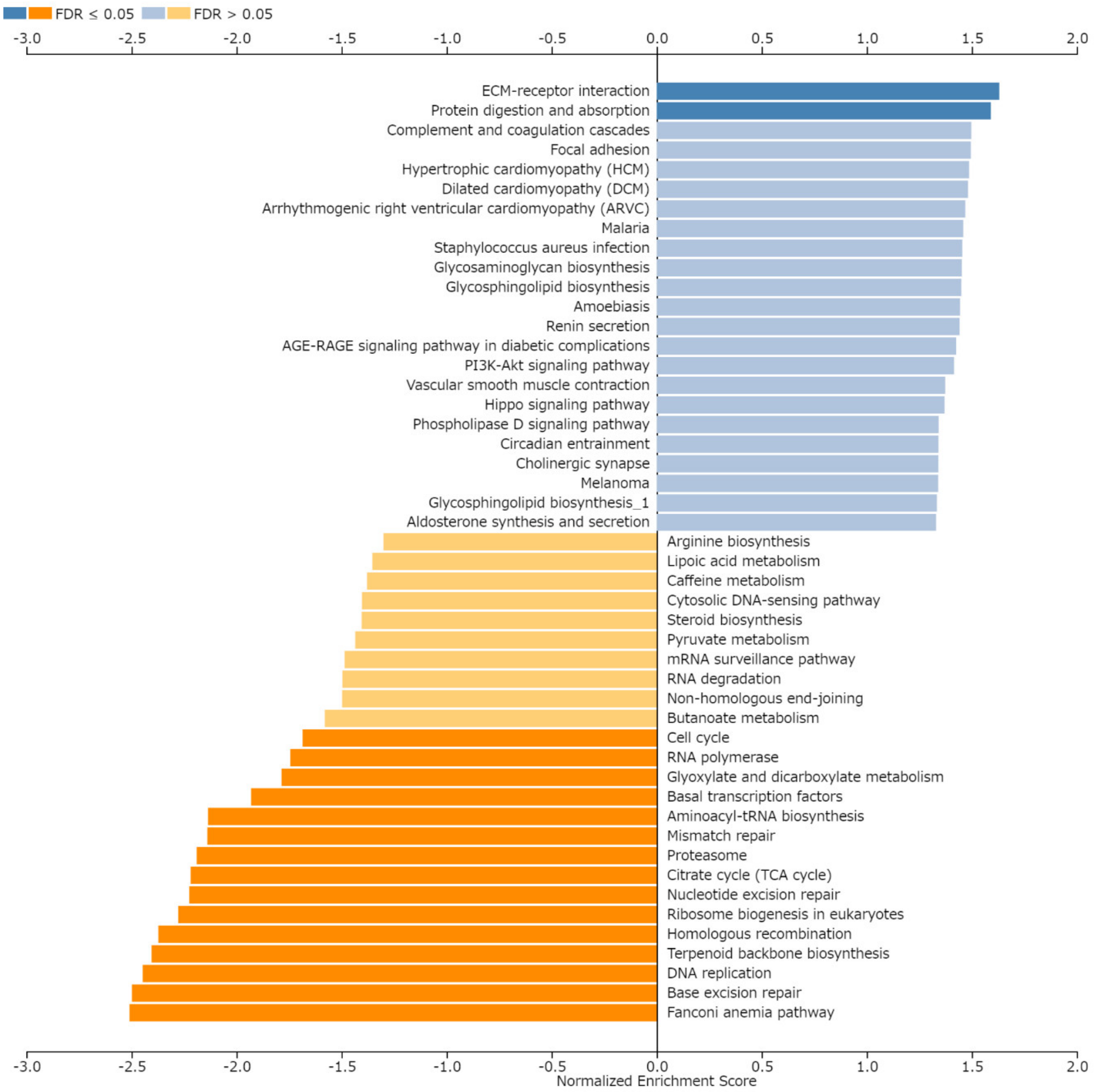

Figure S4

KEGG pathway analysis of genes correlated positively (upper) or negatively (lower) with *AEBP1* using RNA-seq data obtained from primary HNSCCs in a dataset from The Cancer Genome Atlas (TCGA). The analysis was performed using LinkedOmics (http://linkedomics.org).

Figure S5

Figure 2 (F)

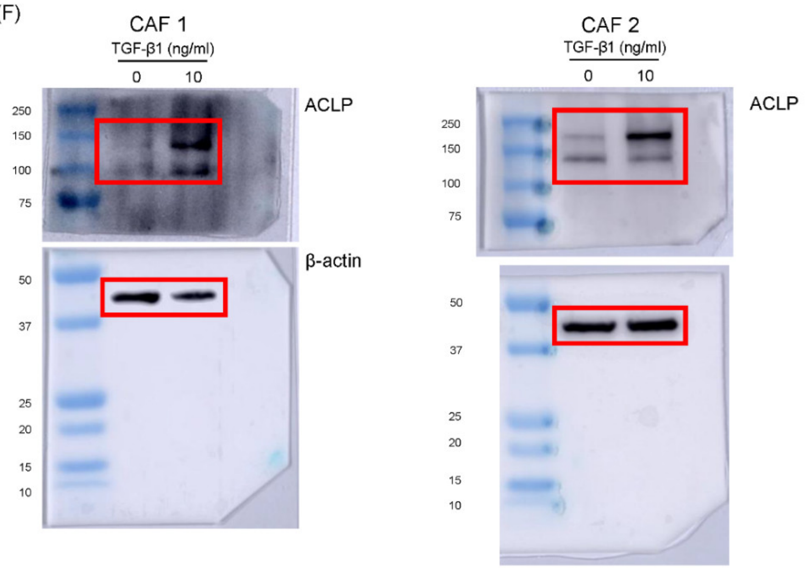

Figure 3 (B)

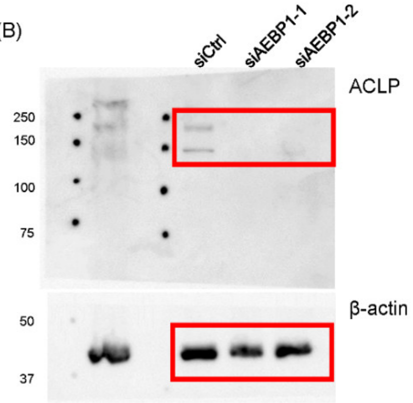

Figure 4 (C)

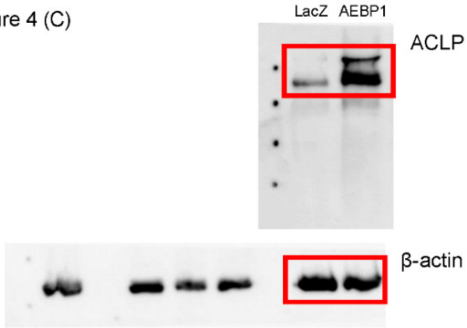

Figure S5

Original images of the western blot analysis.

Figure S6

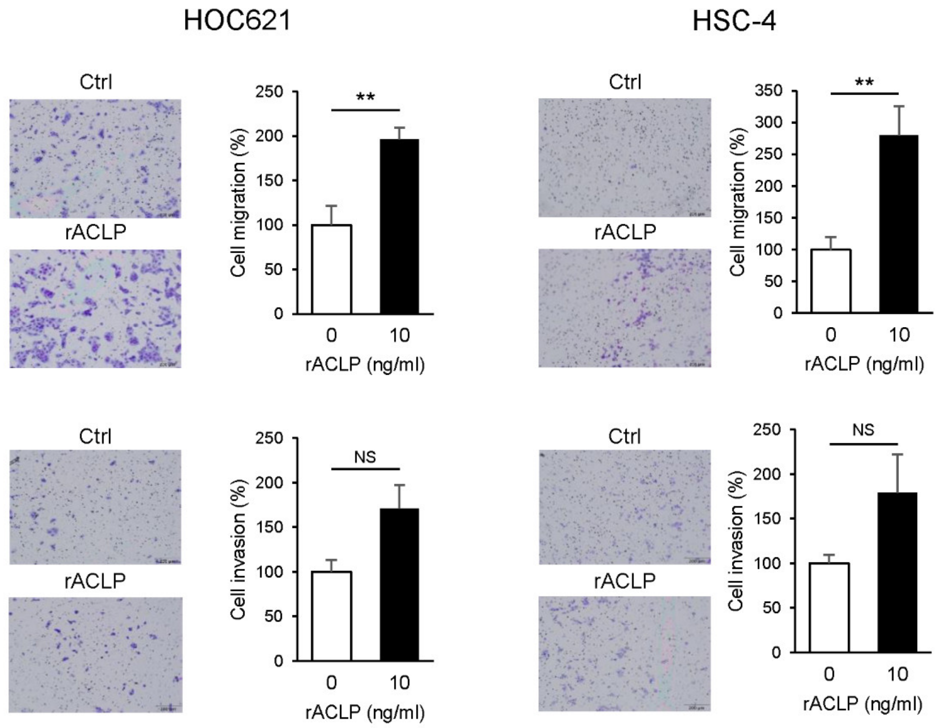

Figure S6

Transwell migration assays using the indicated OSCC cells treated with or without recombinant ACLP. Representative results are shown on the left; summarized results are on the right. (n = 3). Error bars represent SEMs. $^{**}P < 0.01$.

Figure S7

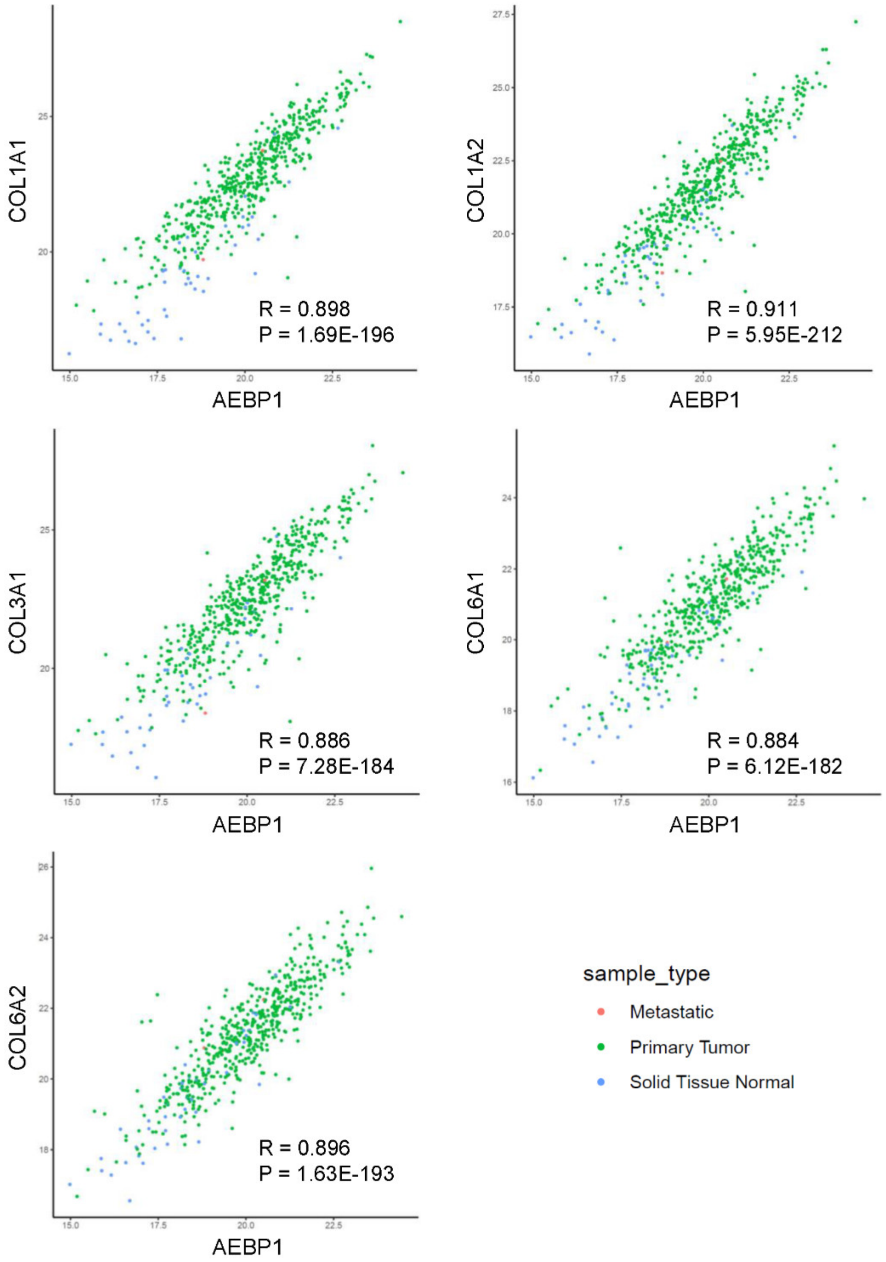

Figure S7

Correlations between mRNA expression levels of the indicated collagen family genes and those of *AEBP1* in primary HNSCCs in a dataset from The Cancer Genome Atlas (TCGA).

Figure S8

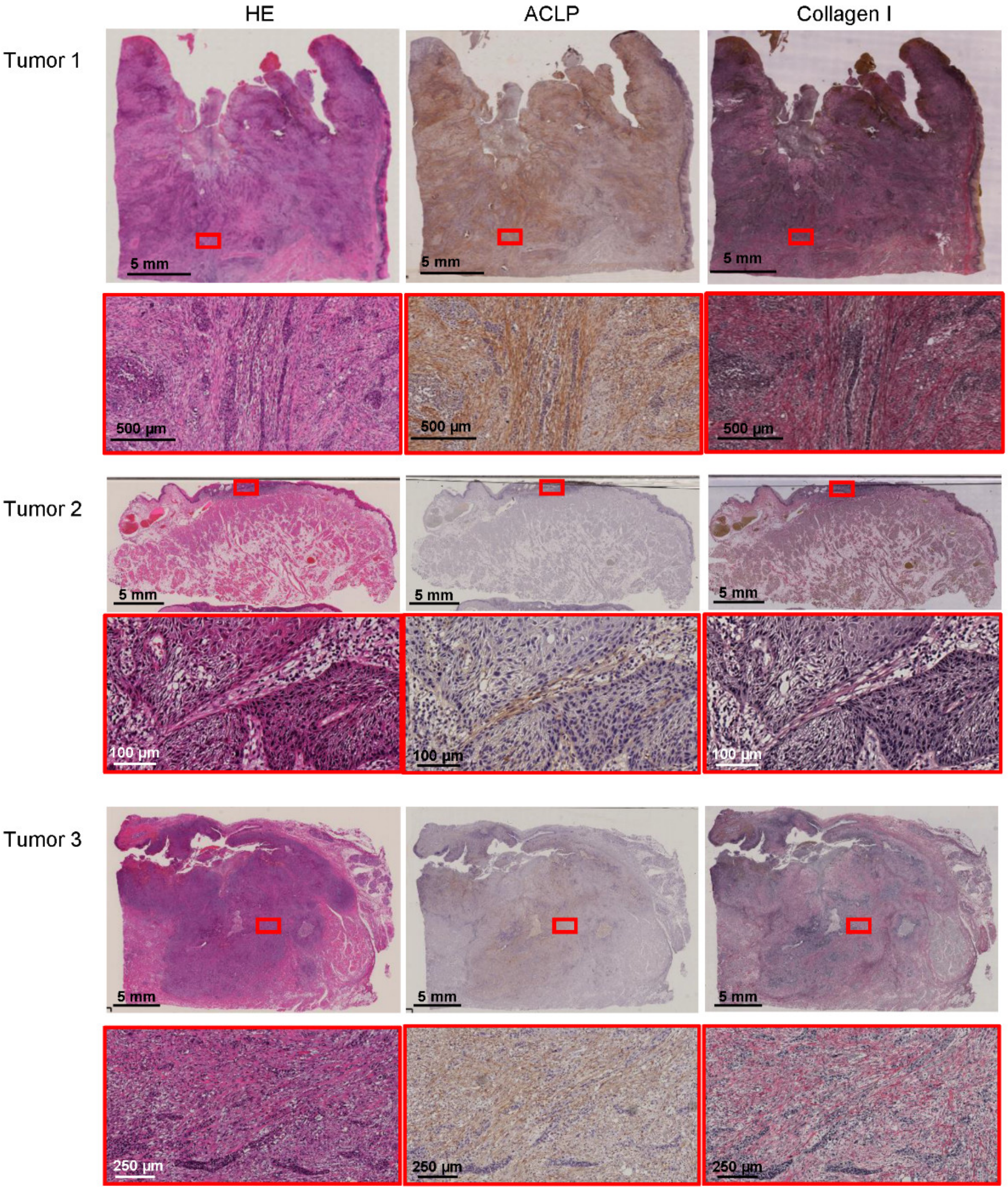

Figure S8

Immunohistochemical staining of ACLP and collagen I in three representative OSCC tissue samples. Magnified views of boxed areas are shown below.

Figure S9

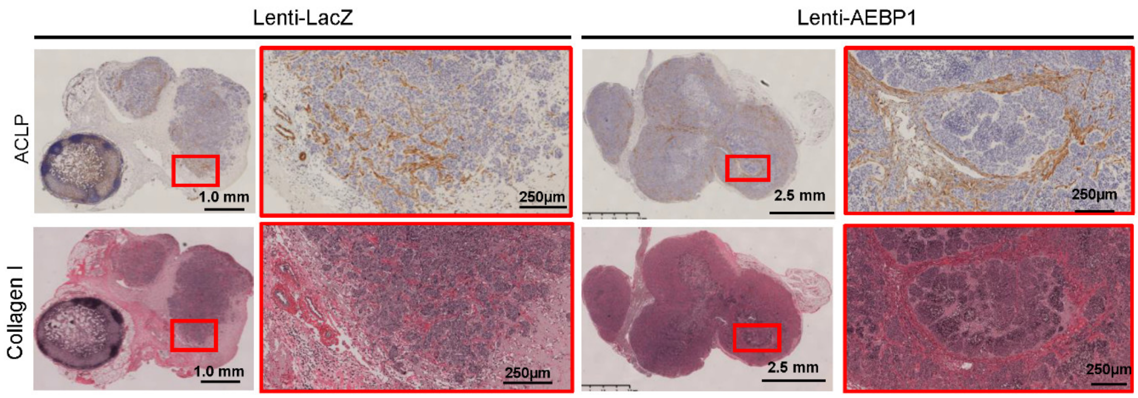

Figure S9

Immunohistochemical staining of ACLP and collagen I in representative xenograft tumors in Figure 5. Mice were transplanted with SAS cells and CAFs (CAF1) infected with the indicated lentiviral vectors.

Figure S10

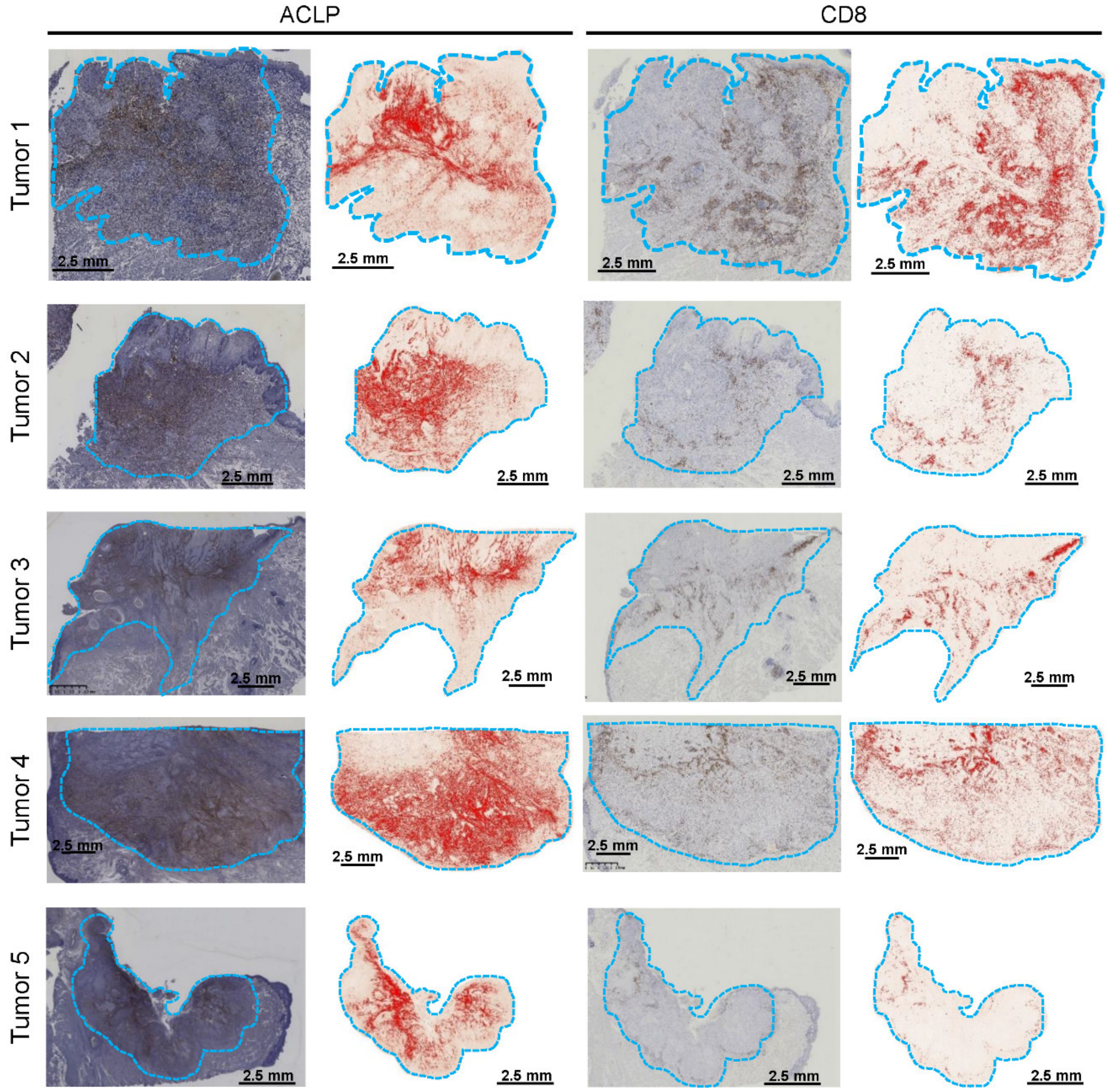

Figure S10

Immunohistochemical staining of ACLP and CD8 in representative OSCC tissues. Blue lines indicate tumor areas, including invasive front regions. Areas positive for ACLP and CD8 are shown on the right.

Table S1. Clinicopathological features of the patients enrolled in this study

		Training set (n = 49)	Validation set (n = 49)
Age (y, mean ± SD)		64.14±10.6	65.29±14.2
Gender	M	37	31
	F	12	18
Tumor size (n, %)	T1, T2	29	47
	T3, T4	20	2
Lymph node metastasis (n, %)	+	11	3
	-	38	46
Stage (n, %)	I, II	32	45
	III, IV	17	4
Differentiation (n, %)	Well-moderate	24	44
	Poor	25	5
Recurrence (n, %)	+	20	18
	-	29	31

Table S2. Sequences of the primers used in this study

RT-PCR		
AEBP1	Forward	5'-TCTTCGTGGGCTTCAGCAATGA-3'
	Reverse	5'-TAGATGCGGATGAAACGAGCCA-3'
	Product size (variant 1)	148 bp
	Product size (variant 2)	251 bp
qRT-PCR		
AEBP1 variant 1	Forward	5'-CATTCTGGCTCCCTCAGAAA-3'
	Reverse	5'-TCTCCTGGATTCTGCCAGTTA-3'
	Product size	120 bp
CDKN1A	Forward	5'-AGACTCTCAGGGTCGAAAACG-3'
	Reverse	5'-TTAGGGCTTCCTCTTGGAGAAG-3'
	Product size	91 bp
VIM	Forward	5'-TGACCTTGAACGCAAAGTGG-3'
	Reverse	5'-TCAGGCTTGGAAACATCCAC-3'
	Product size	138 bp
TWIST	Forward	5'-CCTTCTCGGTCTGGAGGATG-3'
	Reverse	5'-CTGTCCATTTTCTCCTTCTCTGG-3'
	Product size	130 bp
CDH1	Forward	5'-TTTGACGCCGAGAGCTACAC-3'
	Reverse	5'-TCTGTGCCCACTTTGAATCG-3'
	Product size	146 bp
CDH2	Forward	5'-AAGTGGCAGTAAAATTGAGCC-3'
	Reverse	5'-GTGCTTACTGAATTGTCTTGG-3'
	Product size	104 bp
ACTB	Forward	5'-GCCAACCGCGAGAAGATGA-3'
	Reverse	5'-AGCACAGCCTGGATAGCAAC-3'
	Product size	80 bp

Table S3. ACLP expression and clinicopathological features of primary OSCC

		ACLP-low (n = 24)	ACLP-high (n = 25)	P
Age (mean±SD)		64.9±10.3	63.4±10.9	NS
Gender (n, %)	Male	19 (79)	18 (72)	NS
	Female	5 (21)	7 (28)	
Tumor size (n, %)	T1, T2	18 (75)	11 (44)	<0.05
	T3, T4	6 (25)	14 (56)	
Lymph node metastasis (n, %)	+	3 (13)	8 (32)	NS
	-	21 (87)	17 (68)	
Stage (n, %)	I, II	18 (75)	14 (56)	NS
	III, IV	6 (25)	11 (44)	
Differentiation (n, %)	Well, moderate	24 (100)	17 (68)	<0.05
	Poor	0 (0)	8 (32)	
Mode of invasion	YK-2, 3	21 (88)	12 (48)	<0.01
	YK-4C, 4D	3 (12)	13 (52)	
Recurrence (n, %)	+	7 (29)	13 (52)	NS
	-	17 (71)	12 (48)	

Table S4. Top 500 genes co-expressed with AEBP1 in primary HNSCC in TCGA dataset

Gene	R	P
COL1A2	0.9114	5.95E-212
EMILIN1	0.9004	8.03E-199
COL1A1	0.8984	1.69E-196
COL6A2	0.8956	1.63E-193
COL3A1	0.8862	7.28E-184
COL6A1	0.8842	6.12E-182
OLFML2B	0.8820	6.29E-180
COL6A3	0.8786	8.80E-177
PDGFRB	0.8764	9.04E-175
ADAMTS2	0.8749	2.05E-173
PCOLCE	0.8673	5.80E-167
SCARF2	0.8651	3.62E-165
ITGA11	0.8647	8.02E-165
SPARC	0.8644	1.40E-164
TIMP2	0.8612	5.30E-162
THY1	0.8597	7.91E-161
COL5A1	0.8543	1.04E-156
MMP2	0.8538	2.58E-156
VCAN	0.8531	7.41E-156
ADAMTS12	0.8529	1.13E-155
BGN	0.8523	3.33E-155
MXRA8	0.8514	1.50E-154
LAMA4	0.8495	3.60E-153
NOX4	0.8486	1.63E-152
CTSK	0.8467	3.36E-151
POSTN	0.8452	3.58E-150
LRRC15	0.8421	5.78E-148
COL5A2	0.8400	1.44E-146
GLT8D2	0.8371	1.26E-144
BICC1	0.8318	3.48E-141
GGT5	0.8258	2.11E-137
ZNF469	0.8185	4.99E-133
COL8A1	0.8153	3.35E-131
CPXM1	0.8150	5.05E-131
HTRA3	0.8145	9.53E-131
P4HA3	0.8140	1.93E-130
ISLR	0.8119	3.01E-129

KCNE4	0.8047	2.76E-125
FBN1	0.8045	3.36E-125
SGIP1	0.8041	5.62E-125
FN1	0.8039	7.59E-125
ASPN	0.8026	3.50E-124
KIAA1462	0.8003	6.13E-123
HEYL	0.7999	9.86E-123
SULF1	0.7993	1.96E-122
FSTL1	0.7988	3.66E-122
HEPH	0.7987	4.17E-122
CDH11	0.7967	4.42E-121
HHIPL1	0.7925	6.33E-119
RCN3	0.7924	7.38E-119
ADAM12	0.7911	3.12E-118
NTM	0.7904	7.05E-118
FAM198B	0.7904	7.60E-118
FNDC1	0.7893	2.46E-117
COL10A1	0.7889	3.80E-117
SFRP2	0.7872	2.64E-116
TGFB3	0.7872	2.80E-116
OLFML3	0.7860	1.03E-115
CTHRC1	0.7858	1.32E-115
COL11A1	0.7850	3.23E-115
CACNA1C	0.7827	4.11E-114
DACT1	0.7823	6.04E-114
RASGRF2	0.7815	1.53E-113
EFEMP2	0.7792	1.78E-112
CRISPLD2	0.7761	5.05E-111
FAP	0.7749	1.79E-110
THBS2	0.7738	5.87E-110
TMEM119	0.7735	8.16E-110
LRRC32	0.7734	9.14E-110
OLFML1	0.7707	1.55E-108
HIC1	0.7691	7.83E-108
ADAMTS7	0.7677	3.13E-107
SSC5D	0.7631	3.45E-105
CHRD	0.7618	1.17E-104
FBLN2	0.7598	8.44E-104
MMP11	0.7502	8.21E-100
TAGLN	0.7501	8.97E-100

PPAPDC1A	0.7469	1.84E-98
ITGA1	0.7468	1.98E-98
CMTM3	0.7435	4.14E-97
SYDE1	0.7405	5.80E-96
CD248	0.7385	3.41E-95
DACT3	0.7369	1.44E-94
TNFAIP6	0.7365	2.12E-94
FAM26E	0.7349	8.56E-94
NID2	0.7344	1.26E-93
LUM	0.7342	1.49E-93
MYL9	0.7338	2.21E-93
PMP22	0.7308	2.95E-92
ECM2	0.7305	3.74E-92
ZNF521	0.7301	5.04E-92
KIF26B	0.7297	7.29E-92
GPR124	0.7295	8.36E-92
MXRA5	0.7293	1.02E-91
GPC6	0.7293	1.04E-91
CALD1	0.7287	1.74E-91
DDR2	0.7284	2.20E-91
LOXL2	0.7279	3.47E-91
SDC2	0.7262	1.41E-90
KCNJ8	0.7257	2.03E-90
COL12A1	0.7251	3.47E-90
TMEM204	0.7248	4.30E-90
GREM1	0.7247	4.86E-90
RARRES2	0.7231	1.85E-89
PXDN	0.7225	3.07E-89
PCDH12	0.7220	4.37E-89
FMOD	0.7219	4.78E-89
COL18A1	0.7218	5.43E-89
WISP1	0.7216	6.41E-89
CLEC11A	0.7204	1.65E-88
P3H1	0.7190	5.12E-88
PRRX1	0.7188	6.32E-88
CERCAM	0.7178	1.44E-87
COL5A3	0.7175	1.81E-87
ST3GAL2	0.7171	2.41E-87
MMP16	0.7164	4.37E-87
FIBIN	0.7161	5.27E-87

NFATC4	0.7158	7.14E-87
WDR86	0.7156	7.86E-87
KIAA1755	0.7153	1.07E-86
RP11-426C22.4	0.7141	2.72E-86
COLEC12	0.7139	3.16E-86
PDE3A	0.7139	3.16E-86
ADAMTSL2	0.7117	1.72E-85
PLXDC1	0.7113	2.42E-85
PLXND1	0.7105	4.42E-85
MEIS3	0.7105	4.51E-85
CYS1	0.7098	7.66E-85
RAB3IL1	0.7092	1.22E-84
TGFB1I1	0.7087	1.80E-84
COL4A2	0.7054	2.41E-83
WNT2	0.7050	3.09E-83
ANGPTL2	0.7041	6.55E-83
ACTA2	0.7033	1.16E-82
DCHS1	0.7030	1.45E-82
FKBP10	0.7024	2.30E-82
ADAMTS10	0.6983	5.00E-81
CHN1	0.6980	6.37E-81
SFRP4	0.6976	8.50E-81
MRGPRF	0.6974	9.44E-81
FKBP7	0.6962	2.32E-80
GUCY1A2	0.6944	8.78E-80
IGFBP7	0.6943	9.74E-80
TWIST1	0.6931	2.24E-79
DAB2	0.6918	5.93E-79
CSMD2	0.6911	9.45E-79
ANTXR1	0.6903	1.74E-78
FAM19A5	0.6891	3.96E-78
ATP10A	0.6886	5.66E-78
MMP14	0.6874	1.33E-77
LOXL1	0.6867	2.31E-77
ADAMTS14	0.6864	2.70E-77
COL4A1	0.6855	5.31E-77
PDGFRL	0.6849	7.90E-77
CHSY3	0.6831	2.80E-76
LOXL3	0.6829	3.19E-76
ANPEP	0.6816	7.96E-76

LAMP5	0.6813	1.01E-75
GIPC3	0.6806	1.55E-75
ENPP1	0.6805	1.76E-75
SPON1	0.6801	2.19E-75
ZEB2	0.6792	4.26E-75
FAM101A	0.6792	4.26E-75
CNRIP1	0.6784	7.50E-75
LRRC17	0.6783	7.64E-75
STARD13	0.6782	8.36E-75
PTH1R	0.6781	9.13E-75
ADAMTS4	0.6779	1.04E-74
MRC2	0.6775	1.31E-74
UNC5C	0.6770	1.95E-74
LHFP	0.6756	4.77E-74
COL24A1	0.6754	5.51E-74
AMPH	0.6749	8.15E-74
ADAMTS5	0.6743	1.15E-73
JAM3	0.6738	1.63E-73
SYNDIG1	0.6733	2.37E-73
RP11-588K22.2	0.6729	2.94E-73
RP11-863P13.3	0.6710	1.08E-72
VIM	0.6707	1.28E-72
GUCY1A3	0.6706	1.37E-72
GPR4	0.6701	1.92E-72
P3H3	0.6690	4.15E-72
NREP	0.6688	4.78E-72
TBXA2R	0.6679	8.40E-72
GPX8	0.6665	2.14E-71
ENG	0.6650	5.45E-71
NID1	0.6649	5.85E-71
FBLN5	0.6640	1.06E-70
CTD-2171N6.1	0.6636	1.37E-70
HSPA12B	0.6635	1.52E-70
RGS4	0.6627	2.43E-70
AK5	0.6613	6.18E-70
C1QTNF6	0.6606	9.77E-70
TM6SF2	0.6600	1.43E-69
F2R	0.6592	2.31E-69
GALNT15	0.6592	2.33E-69
ACVRL1	0.6591	2.50E-69

HLX	0.6586	3.43E-69
MMP19	0.6577	6.03E-69
DCN	0.6569	1.02E-68
DLC1	0.6560	1.77E-68
SAMD14	0.6560	1.77E-68
PMEPA1	0.6556	2.33E-68
HTRA1	0.6548	3.80E-68
NEGR1	0.6545	4.50E-68
VSTM4	0.6544	4.83E-68
PHLDB1	0.6541	5.78E-68
ZCCHC24	0.6539	6.36E-68
IGF2	0.6522	1.89E-67
CLIP3	0.6520	2.14E-67
GAS1	0.6511	3.63E-67
SOX11	0.6509	4.27E-67
FMNL3	0.6505	5.39E-67
LTBP2	0.6499	7.88E-67
NTNG2	0.6498	8.07E-67
CACNA1H	0.6480	2.42E-66
DNM3OS	0.6476	3.09E-66
MAGEL2	0.6465	5.97E-66
EBF1	0.6460	8.23E-66
BNC2	0.6459	8.73E-66
LAMB1	0.6446	1.92E-65
PLVAP	0.6436	3.53E-65
APBA2	0.6433	4.30E-65
RP11-426C22.5	0.6431	4.79E-65
AP000892.6	0.6429	5.41E-65
SERPINH1	0.6427	6.13E-65
FGFR1	0.6426	6.25E-65
FERMT2	0.6425	6.95E-65
MSRB3	0.6424	7.03E-65
PDGFRA	0.6421	8.42E-65
RUNX1T1	0.6416	1.18E-64
ENPEP	0.6415	1.21E-64
MAP1A	0.6412	1.50E-64
ZEB1	0.6405	2.22E-64
ZNF423	0.6396	3.89E-64
SGCD	0.6391	5.09E-64
SHANK1	0.6369	1.88E-63

PODN	0.6367	2.09E-63
GLIS3	0.6366	2.16E-63
FYN	0.6358	3.57E-63
CTGF	0.6357	3.78E-63
TCF4	0.6329	1.94E-62
CREB3L1	0.6314	4.54E-62
FBXL7	0.6310	5.79E-62
EDNRA	0.6304	7.94E-62
PCDH18	0.6297	1.20E-61
SEPT4	0.6286	2.26E-61
SYT11	0.6285	2.40E-61
SH3RF3	0.6277	3.78E-61
ARHGEF17	0.6275	4.02E-61
CASC15	0.6274	4.33E-61
C3orf80	0.6269	5.89E-61
PLXNC1	0.6260	9.73E-61
DAAM2	0.6253	1.42E-60
TIE1	0.6242	2.60E-60
TNS3	0.6241	2.81E-60
GLIS2	0.6238	3.29E-60
LRCH2	0.6238	3.35E-60
DLL4	0.6226	6.26E-60
MFAP2	0.6216	1.11E-59
DOK6	0.6213	1.33E-59
FOXS1	0.6196	3.35E-59
SNAI1	0.6192	4.21E-59
GPR173	0.6186	5.91E-59
TMEM26	0.6186	5.93E-59
CNN1	0.6185	6.13E-59
RP11-576I22.2	0.6171	1.30E-58
GUCY1B3	0.6170	1.37E-58
ARSB	0.6155	3.09E-58
WIPF1	0.6154	3.28E-58
VASH1	0.6152	3.61E-58
LINC01561	0.6151	3.98E-58
HMCN1	0.6144	5.65E-58
VGLL3	0.6132	1.09E-57
CPQ	0.6128	1.36E-57
NOVA2	0.6123	1.73E-57
ARHGEF40	0.6118	2.24E-57

MICAL2	0.6112	3.16E-57
CLSTN2	0.6109	3.60E-57
FGD5	0.6107	4.04E-57
LAMA2	0.6100	5.92E-57
ITGBL1	0.6099	6.36E-57
GLIS1	0.6097	7.11E-57
COL8A2	0.6096	7.21E-57
COL15A1	0.6078	1.91E-56
RASA3	0.6067	3.42E-56
ARHGEF25	0.6065	3.66E-56
SPON2	0.6065	3.76E-56
CCDC102B	0.6063	4.12E-56
C1QTNF1	0.6059	5.16E-56
PCDH17	0.6055	6.23E-56
LAMB2	0.6044	1.11E-55
DSEL	0.6041	1.28E-55
SEMA6B	0.6034	1.90E-55
CORIN	0.6023	3.28E-55
DGKI	0.6022	3.53E-55
MME	0.6004	9.03E-55
SLC24A2	0.6001	1.04E-54
IGDCC4	0.5991	1.69E-54
DIRC1	0.5988	1.98E-54
FBLN7	0.5982	2.72E-54
RECK	0.5975	3.96E-54
GXYLT2	0.5974	4.14E-54
SPOCK1	0.5971	4.86E-54
AXL	0.5967	5.82E-54
RHOJ	0.5956	1.00E-53
ADAMTS16	0.5952	1.22E-53
RPLP0P2	0.5946	1.68E-53
ANXA6	0.5942	2.04E-53
CCDC80	0.5939	2.34E-53
RBPMS2	0.5936	2.78E-53
PPEF1	0.5931	3.57E-53
TMEM200A	0.5930	3.73E-53
A2M	0.5909	1.07E-52
ITGA5	0.5908	1.14E-52
MMP9	0.5907	1.16E-52
CD93	0.5904	1.34E-52

DCLK2	0.5892	2.46E-52
ST8SIA2	0.5889	2.83E-52
FILIP1L	0.5889	2.87E-52
MAGI2-AS3	0.5881	4.26E-52
CNPY4	0.5875	5.65E-52
GRID1	0.5873	6.13E-52
IGFBP4	0.5872	6.63E-52
RHOBTB1	0.5867	8.29E-52
NNMT	0.5862	1.09E-51
STK32B	0.5860	1.20E-51
PRKG1	0.5852	1.75E-51
CYGB	0.5849	2.03E-51
FZD2	0.5843	2.74E-51
GPR116	0.5834	4.18E-51
TTYH3	0.5831	4.89E-51
TNN	0.5819	8.40E-51
CALU	0.5819	8.46E-51
C1S	0.5811	1.26E-50
MATN3	0.5811	1.27E-50
TBX2	0.5802	1.90E-50
KCND2	0.5801	2.00E-50
MSC	0.5799	2.27E-50
RP5-952N6.1	0.5795	2.65E-50
TUBA1A	0.5792	3.12E-50
SLC41A2	0.5792	3.15E-50
PTK7	0.5791	3.23E-50
S1PR2	0.5783	4.84E-50
MRAS	0.5774	7.14E-50
LAMC1	0.5773	7.58E-50
AC093850.2	0.5768	9.84E-50
ENTPD1	0.5764	1.19E-49
TMEM47	0.5762	1.31E-49
PTPRN	0.5760	1.43E-49
TMEM255B	0.5747	2.60E-49
FLT4	0.5732	5.14E-49
APLNR	0.5724	7.49E-49
ADAMTS6	0.5723	8.08E-49
CLMP	0.5716	1.11E-48
ACAN	0.5715	1.16E-48
ATP8B2	0.5702	2.07E-48

PDGFB	0.5700	2.32E-48
KDELR3	0.5693	3.24E-48
TSHZ3	0.5692	3.32E-48
C11orf96	0.5687	4.28E-48
SLC2A3	0.5682	5.36E-48
RP5-907D15.4	0.5675	7.27E-48
MRVI1	0.5672	8.32E-48
GSC	0.5672	8.33E-48
BMP8A	0.5670	9.21E-48
PTGIR	0.5663	1.28E-47
F2RL2	0.5655	1.78E-47
ARSE	0.5648	2.47E-47
DLG4	0.5646	2.73E-47
EDIL3	0.5644	3.05E-47
MAGEH1	0.5635	4.58E-47
RP5-1059L7.1	0.5634	4.62E-47
RASL12	0.5631	5.35E-47
SERPING1	0.5625	6.98E-47
TMEM130	0.5619	9.23E-47
ELTD1	0.5618	9.51E-47
PTPRM	0.5616	1.07E-46
BCL6B	0.5611	1.31E-46
C1R	0.5604	1.83E-46
ADAMTSL1	0.5602	1.99E-46
AKAP12	0.5601	2.06E-46
MARVELD1	0.5594	2.82E-46
NPR2	0.5585	4.11E-46
AC004538.3	0.5575	6.58E-46
RUNX2	0.5572	7.40E-46
TNFSF4	0.5571	7.75E-46
LDB2	0.5568	8.84E-46
TSPAN11	0.5559	1.34E-45
CDH6	0.5558	1.38E-45
RNF144A-AS1	0.5555	1.55E-45
COMP	0.5554	1.67E-45
SH3PXD2B	0.5547	2.21E-45
PIEZO2	0.5534	3.98E-45
BMP1	0.5531	4.57E-45
RP11-513O13.1	0.5527	5.39E-45
GPR162	0.5526	5.69E-45

CALHM2	0.5521	6.91E-45
ARMC9	0.5519	7.55E-45
ITGB5	0.5517	8.41E-45
LINC00922	0.5515	8.88E-45
PNMA2	0.5505	1.38E-44
SCG2	0.5502	1.57E-44
ALPK2	0.5499	1.83E-44
IGF2-AS	0.5492	2.45E-44
NKX3-2	0.5491	2.56E-44
GRIA3	0.5486	3.15E-44
PEAK1	0.5481	3.83E-44
MIR143HG	0.5480	4.10E-44
F13A1	0.5480	4.14E-44
RP11-417E7.2	0.5479	4.31E-44
HEG1	0.5474	5.22E-44
NKD1	0.5470	6.28E-44
TENM3	0.5469	6.58E-44
ZFP92	0.5465	7.75E-44
NUAK1	0.5460	9.35E-44
CSGALNACT2	0.5456	1.14E-43
ABCC9	0.5456	1.14E-43
FAM101B	0.5446	1.70E-43
INHBA	0.5445	1.76E-43
ACE	0.5445	1.80E-43
AP001471.1	0.5444	1.85E-43
LRP1	0.5441	2.17E-43
RP11-13P5.2	0.5425	4.19E-43
NAP1L3	0.5423	4.55E-43
KANK2	0.5414	6.74E-43
ST6GALNAC5	0.5413	6.89E-43
ENOX1	0.5411	7.67E-43
FZD1	0.5409	8.18E-43
AC007750.5	0.5403	1.06E-42
FLT1	0.5400	1.17E-42
EVC	0.5395	1.48E-42
CTD-3247H4.2	0.5393	1.56E-42
EPDR1	0.5390	1.82E-42
LHFPL2	0.5389	1.91E-42
CD276	0.5384	2.36E-42
LPAR4	0.5378	2.94E-42

GFPT2	0.5377	3.07E-42
LSAMP	0.5373	3.70E-42
CTSZ	0.5372	3.76E-42
JAM2	0.5360	6.23E-42
GALNT10	0.5359	6.55E-42
FAM225B	0.5358	6.80E-42
MSR1	0.5356	7.35E-42
NRP1	0.5355	7.69E-42
GJC1	0.5333	1.91E-41
STARD8	0.5331	2.03E-41
CDH2	0.5330	2.12E-41
AKT3	0.5330	2.14E-41
PRICKLE1	0.5330	2.15E-41
SCARF1	0.5328	2.31E-41
OMD	0.5326	2.46E-41
LINC01048	0.5325	2.61E-41
KIRREL	0.5323	2.87E-41
NRXN2	0.5317	3.53E-41
TRPV2	0.5312	4.35E-41
AGTR1	0.5310	4.71E-41
RGS16	0.5310	4.79E-41
PLBD2	0.5309	4.92E-41
PTGDR	0.5309	4.94E-41
PDE1B	0.5306	5.56E-41
MMP13	0.5305	5.90E-41
TIMP1	0.5304	6.17E-41
EPHA3	0.5303	6.42E-41
AMPD2	0.5302	6.71E-41
LIN7A	0.5296	8.25E-41
ESAM	0.5286	1.24E-40
CXorf36	0.5284	1.39E-40
COL16A1	0.5279	1.64E-40
FAM155A	0.5278	1.72E-40
TRO	0.5277	1.78E-40
LINC00654	0.5276	1.84E-40
BST1	0.5272	2.21E-40
IFFO1	0.5272	2.21E-40
PPFIA2	0.5268	2.61E-40
LDLRAD4	0.5265	2.88E-40
LINGO1	0.5264	3.04E-40

C16orf45	0.5263	3.13E-40
MFGE8	0.5259	3.72E-40
C5AR1	0.5256	4.08E-40
KANK4	0.5251	5.04E-40
APBB2	0.5251	5.08E-40
FBLN1	0.5248	5.74E-40
RP11-554A11.4	0.5245	6.54E-40
CDH5	0.5242	7.17E-40
RCAN2	0.5241	7.51E-40
GPX7	0.5237	8.72E-40
NOTCH4	0.5231	1.11E-39
RP11-175K6.2	0.5231	1.12E-39
PKIG	0.5227	1.29E-39
ROR2	0.5225	1.39E-39
SUSD2	0.5225	1.43E-39
C14orf37	0.5222	1.58E-39
SPOCD1	0.5220	1.74E-39
RFX8	0.5215	2.12E-39
MCAM	0.5213	2.28E-39
CCIN	0.5213	2.30E-39
EVA1B	0.5211	2.44E-39
S1PR3	0.5209	2.64E-39
FKBP9	0.5206	3.03E-39
